# Parallel odor processing by mitral and middle tufted cells in the olfactory bulb

**DOI:** 10.1038/s41598-018-25740-x

**Published:** 2018-05-16

**Authors:** Francesco Cavarretta, Shawn D. Burton, Kei M. Igarashi, Gordon M. Shepherd, Michael L. Hines, Michele Migliore

**Affiliations:** 1 Department of Neuroscience, Yale University School of Medicine, 06510 New Haven, CT USA; 2 Dipartimento di Matematica “Federigo Enriques”, Universita’ degli Studi di Milano, 20133 Milano, Italy; 3 Department of Biological Sciences, Carnegie Mellon University, 15213 Pittsburgh, PA USA; 4 Center for the Neural Basis of Cognition, University of Pittsburgh, 15213 Pittsburgh, PA USA; 5 Department of Neurobiology and Anatomy, University of Utah, 84112 Salt Lake City, UT USA; 6 Department of Anatomy and Neurobiology, and Center for the Neurobiology of Learning and Memory, University of California, 92697 Irvine, CA USA; 7 Institute of Biophysics, National Research Council, 90146 Palermo, Italy

## Abstract

The olfactory bulb (OB) transforms sensory input into spatially and temporally organized patterns of activity in principal mitral (MC) and middle tufted (mTC) cells. Thus far, the mechanisms underlying odor representations in the OB have been mainly investigated in MCs. However, experimental findings suggest that MC and mTC may encode parallel and complementary odor representations. We have analyzed the functional roles of these pathways by using a morphologically and physiologically realistic three-dimensional model to explore the MC and mTC microcircuits in the glomerular layer and deeper plexiform layer. The model makes several predictions. MCs and mTCs are controlled by similar computations in the glomerular layer but are differentially modulated in deeper layers. The intrinsic properties of mTCs promote their synchronization through a common granule cell input. Finally, the MC and mTC pathways can be coordinated through the deep short-axon cells in providing input to the olfactory cortex. The results suggest how these mechanisms can dynamically select the functional network connectivity to create the overall output of the OB and promote the dynamic synchronization of glomerular units for any given odor stimulus.

## Introduction

The aim of this study is to understand how olfactory bulb (OB) microcircuits process odor inputs as the first step toward olfactory perception. It is well established that the OB transforms sensory input from the olfactory sensory neurons (OSNs) into spatially and temporally organized activity patterns in the olfactory glomerular layer^1–5^. Neural circuits in the OB process these patterns and send them to the olfactory cortex (OC), which carries out higher association processing^6–9^ for further output to the orbitofrontal cortex. Understanding how information is transformed in the OB and outputted to the OC is therefore critical for understanding the higher association basis of olfactory perception.

This transformation is mediated by two populations of principal neurons, the mitral (MC) and middle tufted (mTC) cells^10^. Until now nearly all studies of odor processing have focused on the MCs or on undifferentiated MC/tufted cells, leaving the role of the mTCs largely unexplored. Experimental studies of these cells have been limited by their smaller size and less distinct laminar organization. There are at present no recordings of the two populations of cells responding simultaneously to natural odor inputs. It is urgent therefore to characterize the mTC properties, show their role in odor processing, and postulate how the MC and mTC pathways work in parallel to guide further experiments.

In addressing a complex problem such as this when experimental data is limited, biophysically and morphologically realistic computational models have been found to be valuable in providing insights that can guide future experiments. We have previously used this approach to build scaled-up models of MCs and their associated microcircuits^11–13^. The latest version of the model has included 635 MCs and about 100,000 granule cells (GC), together with circuits mediating intra- and inter-glomerular interactions. The OB thus joins the hippocampus CA1^14^ and the neocortex^15^ as realistic data-driven simulations of large populations of interacting forebrain cortical neurons.

Here we extend the OB model to include the mTCs. The results point toward a much expanded view of OB processing. The MCs and mTCs are controlled in similar ways by glomerular layer circuitry, but become separate at the GC level. We show that the intrinsic electrophysiological properties of mTCs are adapted to promote their synchronization through a common GC input. Deep short-axon cells (dSAC) are postulated to play a key role in coordinating the output at the GC level. Specific connections are tested that could provide the mechanisms of coordination. Overall the results suggest that mTCs exert critical control over the output of the OB, promoting in specific ways the functional and dynamic synchronization of ensembles of MC and mTCs.

## Results

**Figure 1:**
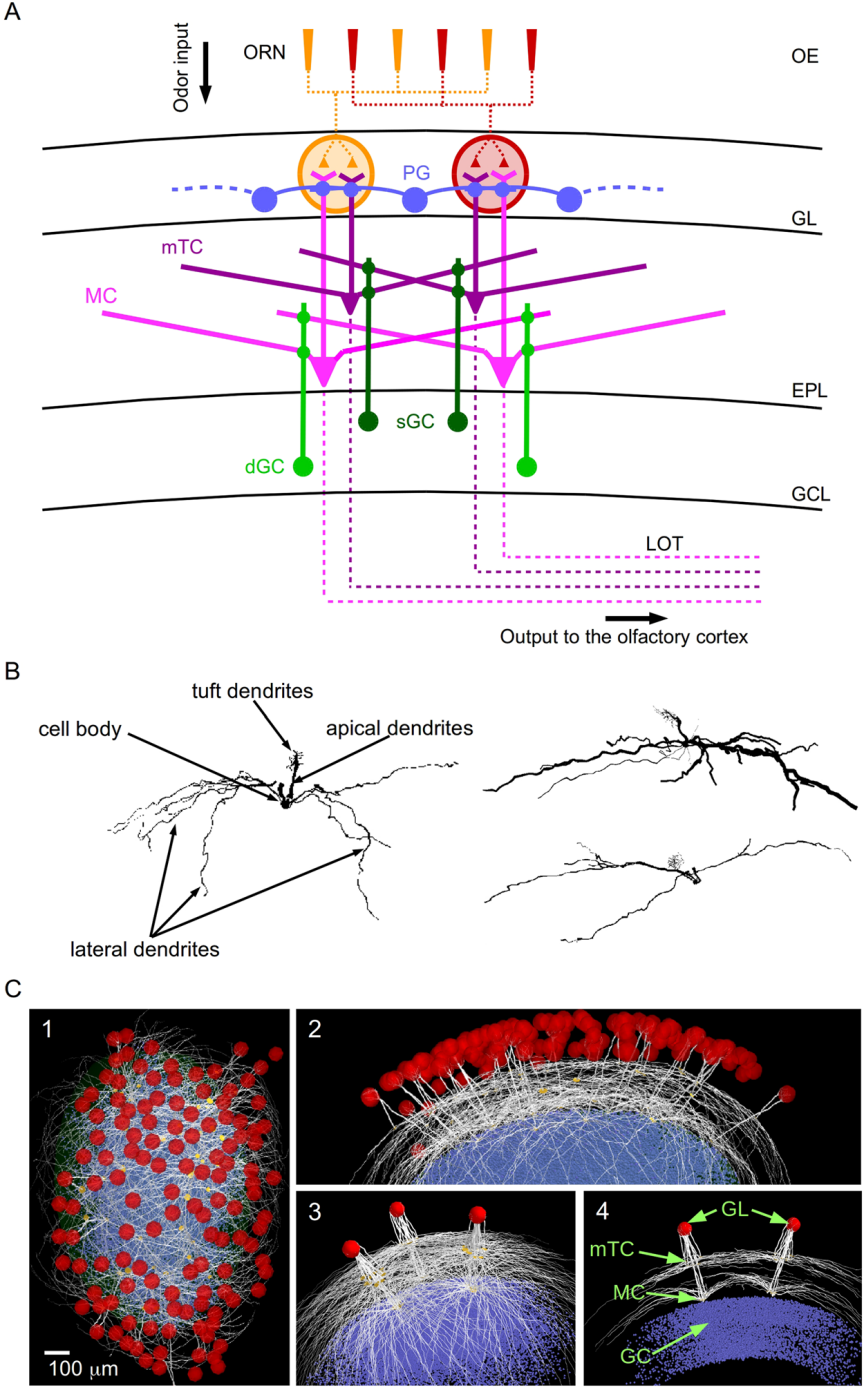
Schematic representation of the olfactory bulb. (**A**) The translaminar organization of the olfactory bulb (OB) and its position in the olfactory pathway. The olfactory sensory neurons (OSNs) in the olfactory epithelium (OE) project to the glomerular cell layer (GL) of the OB, where they connect to periglomerular (PGC), as well as mitral (MC) and middle tufted (mTC) cells, which connect to granule (GC) cells; the model also implicitly takes into account the effect of external tufted cells (ETC) (27, 57, 78), not shown in the diagram. Laminae: glomerular layer (GL); external plexiform layer (EPL); granule cell layer (GCL). The OB output propagates to the olfactory cortex (OC) through the MC and mTC axons in the lateral olfactory tract (LOT). (**B**) Three full mTC reconstructions. (**C**) Full model seen en face (1) and from the side (2) (glomeruli in red); 3: three typical glomerular units, formed by MCs, mTCs, and GCs; 4: slice cut illustrating the different spatial organizations of the dendritic arborizations of MCs and mTCs within the EPL.

We started from the large scale, experimentally-constrained, realistic three-dimensional (3D) model of the OB mentioned in the Introduction^13^. As shown in Fig. 1A, this involved the MCs (light magenta) receiving input from the OSNs (red and yellow) in their distal apical tufts, the GCs (light green), and the glomerular layer interactions mediated by periglomerular cells (PGCs, blue) and external tufted cells (ETCs) (not shown in Fig. 1A for clarity). With this model we have shown how the MCs interact with a system of intra- and inter-glomerular mechanisms that shape their odor responses^13^. The glomerular layer conveys feedforward inhibition to the MC tuft dendrites, modulating the sensory input conveyed from the OSNs. Deeper in the OB, the MC lateral dendrites interact with GC dendrites to bring about feedback and lateral inhibition. Both feedforward and feedback inhibition thus shape the MC output to the olfactory cortex.

Here we expand the model by adding the mTCs (Fig. 1A, dark purple), which have their connections in the glomerular layer, and their population of superficial GCs (sGC, Fig. 1A, dark green) contrasted with the deep GCs (dGC, Fig. 1A, light green) interacting with MCs. The mTCs were synthesized from 6 full 3D reconstructions^16^ (see *Methods*), three of which are shown in Fig. 1B. A 3D rendering of the new full model is shown in Fig. 1C1,C2, with only one MC and mTC cell per glomerulus for the sake of clarity. Figure 1C3 shows three glomeruli with the full set of MCs and mTCs projecting to them; note their highly overlapping lateral dendrites. A 150 µm slice (Fig. 1C4) gives a better representation of the internal layout of the model. More details are given in *Methods*.

### The different membrane properties of mitral and middle tufted cells

Having set up the morphology and connectivity in the expanded model, we next simulated the functional properties of MCs and mTCs. The first experimental constraint was the response to somatic current injection (Fig. S1). MCs responded with tonic firing, whereas mTCs responded with high frequency bursting at low intensities, changing to high frequency tonic firing at high intensities, consistent with experiment^17^. We explored the functional consequences of the different intrinsic properties with a stimulation protocol including hyperpolarizing pulses, which resulted in robust rebound bursts only in the mTCs.

**Figure 2:**
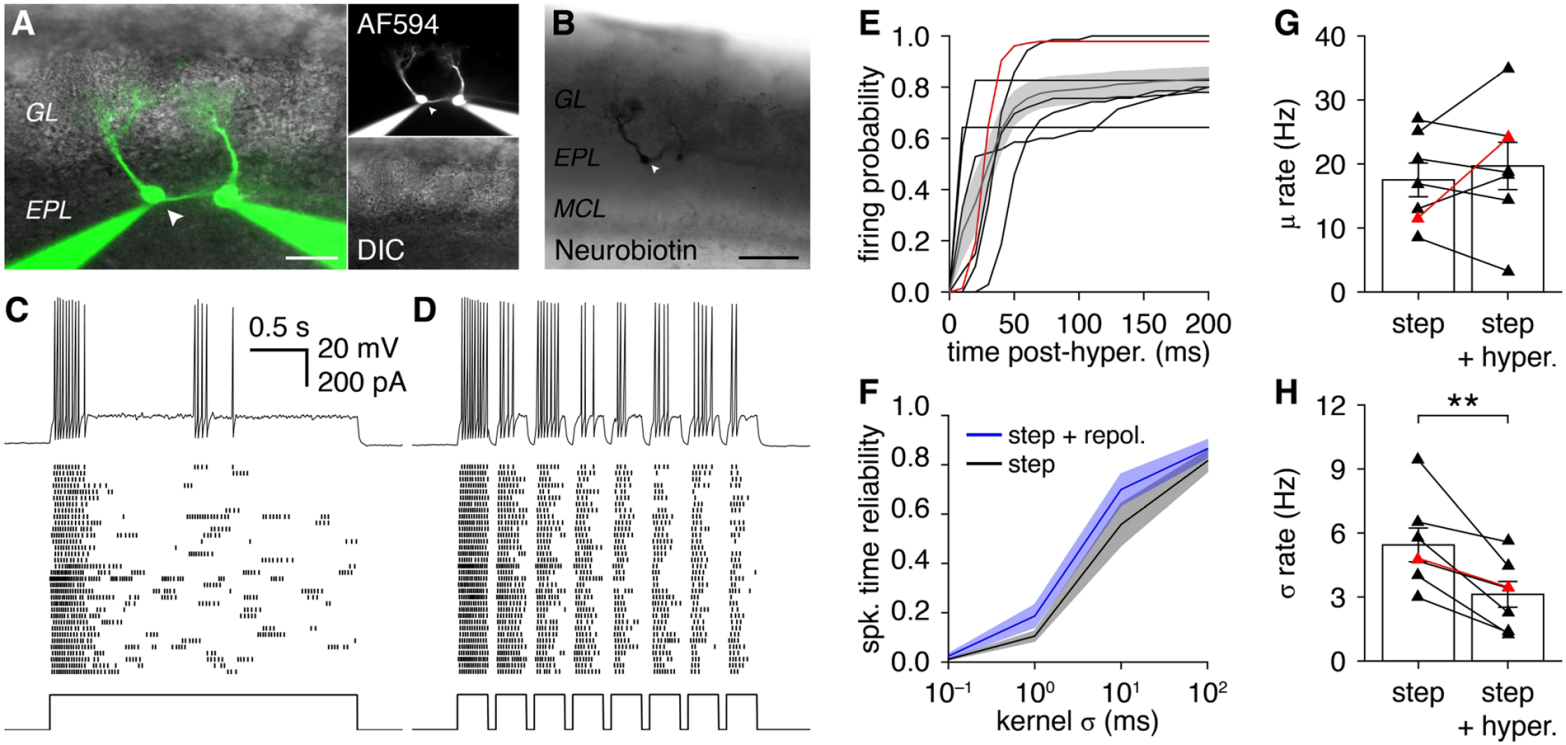
Repolarization enhances middle tufted cell firing reliability. (**A**,**B**) Whole-cell recording of a representative pair of mTCs, showing intracellular fills with AF594 (**A**) and Neurobiotin (**B**). Scale bar: 20 μm (**A**); 100 μm (**B**). (**C**,**D**) Firing patterns evoked in the representative mTC shown in (**A**,**B**) (arrowhead) evoked by constant step current injection (**C**, lower) or constant step current injection with 50 ms-pauses in depolarization (**D**, lower), mimicking the hyperpolarization pulses simulated in Fig. S1. A single voltage trace is shown (top), with a raster plot of spike times across multiple trials below (voltage trace corresponds to first trial in raster plot). Constant step current injection evokes unreliable clusters of action potentials (**C**), while intermittent hyperpolarizations (such as from inhibitory inputs) evokes reliable clusters of action potentials (**D**). (**E**) Mean probability of mTC firing following repolarization (n = 7). Black lines correspond to different mTCs; red line corresponds to representative mTC shown in (**A**–**D**). Average first-spike latency following repolarization: 30.2 ± 18.3 ms. (**F**) Mean trial-to-trial spike-time reliability (n = 7), calculated from Gaussian-convolved spike trains with varying kernel σ^74^. Intermittent repolarizations enhance reliability across a large range of kernel σ, with reliability at small σ indicative of fast timescale synchrony and reliability at large σ indicative of slow timescale firing rate correlation. (**G**) Intermittent repolarizations evoked rapid firing (**D**,**E**) but also frequently terminated firing, yielding an insignificant net change in spike-count firing rates (p = 0.44, paired t test; n = 7). (**H**) Intermittent repolarizations significantly reduced trial-to-trial variation in spike-count firing rates (p = 5.7 × 10^−3^, paired t test; n = 7), consistent with enhanced spike-time reliability at large σ (**F**).

To test this model, we carried out experiments in acute slices (Fig. 2A,B). mTCs at resting membrane potential responded to constant step current depolarizations with an irregular firing of action potential clusters and subthreshold oscillations (Fig. 2C), as previously observed^17^. Introducing brief (50 ms) pauses in depolarization (i.e., 0 pA injection), to mimick hyperpolarizing pulses, rapidly and reliably evoked additional action potential clusters (Fig. 2D,E), consistent with the rebound bursts observed in our model (Fig. S1). Such rebound burst activity robustly enhanced trial-to-trial spike-time reliability on both fast timescales (i.e., spike synchrony) and slow timescales (i.e., firing rate correlations) (Fig. 2F,H). These results suggest that mTCs, which exhibit more irregular firing of action potential clusters than MCs^17^, may be preferentially synchronized by phasic hyperpolarization via synaptic inhibition.

**Figure 3:**
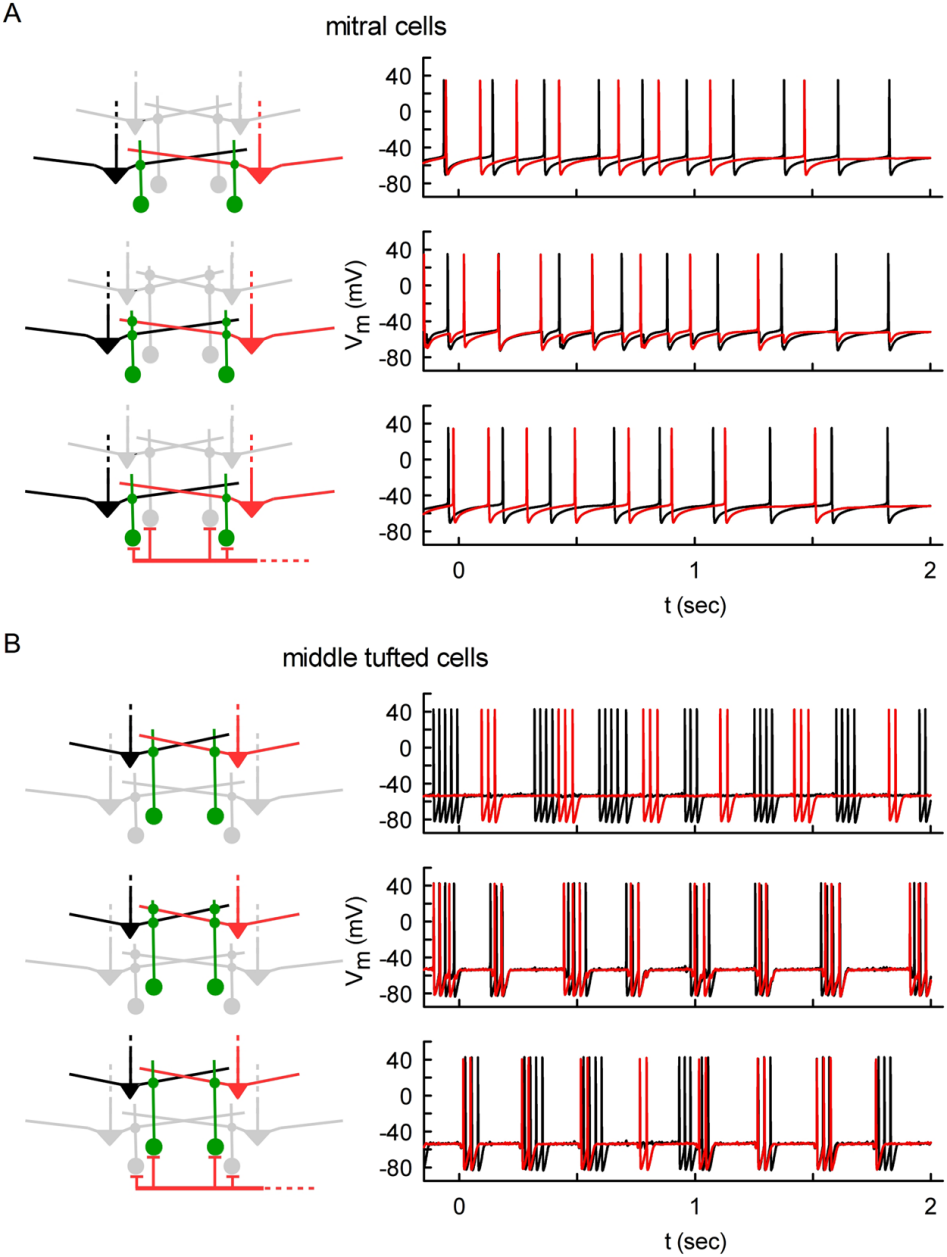
Instantaneous and robust synchronization in middle tufted cells via granule cells. Firing in two MCs (**A**) or mTCs (**B**) that are connected to two GCs with different configurations of connectivity. MCs and mTCs have connections (green dots) to different GCs (top), reciprocal connections (middle), and separate GCs on which axons from the OC provide the same excitatory input (bottom). Note that only mTCs were robustly synchronized in the latter two cases, as described in the text.

We next studied the consequences of this rebound burst activity. Its occurrence suggested that mTCs could be synchronized following a common GC input, which could come from GCs that connect to MCs and mTCs. Experimental findings showed that GCs interact with mTCs and MCs connecting exclusively to different glomeruli^18^. We therefore explored the possibility of a synchronous rebound burst, triggered by common GC input to two mTCs connected to different glomeruli. For this purpose, we simulated two MCs (Fig. 3A) or two mTCs (Fig. 3B) connected to two GCs (green dots, see the wiring diagrams). We used only two GCs to take into account the experimental evidence that the majority of GCs are silent, in part due to tonic inhibition by dSACs^19^. The system was weakly activated at 350 ms intervals by an input reproducing the experimentally observed dynamics for glomerular activity during an odor presentation with ~3 Hz sniffing^20^. Note that even when the two MCs (Fig. 3A,B, top panels, black and red traces) were activated in the same way, they tended to fire asynchronously because of the physiologically-significant different morphologies. We then investigated different configurations of connectivity. As can be seen, MC firing was not significantly affected by the feedback and lateral inhibition due to GCs (Fig. 3A), and their firing did not differ despite different types of connectivity. In contrast, mTCs were instantaneously synchronized by the GC inhibition when they were reciprocally connected through GCs (Fig. 3B, middle panels), or when the GCs received external synchronous excitation, even in the absence of reciprocal connections (Fig. 3B, bottom panels). There is experimental evidence suggesting that this external excitation may be mediated through centrifugal fibers projecting from the cortex^21–24^.

**Figure 4:**
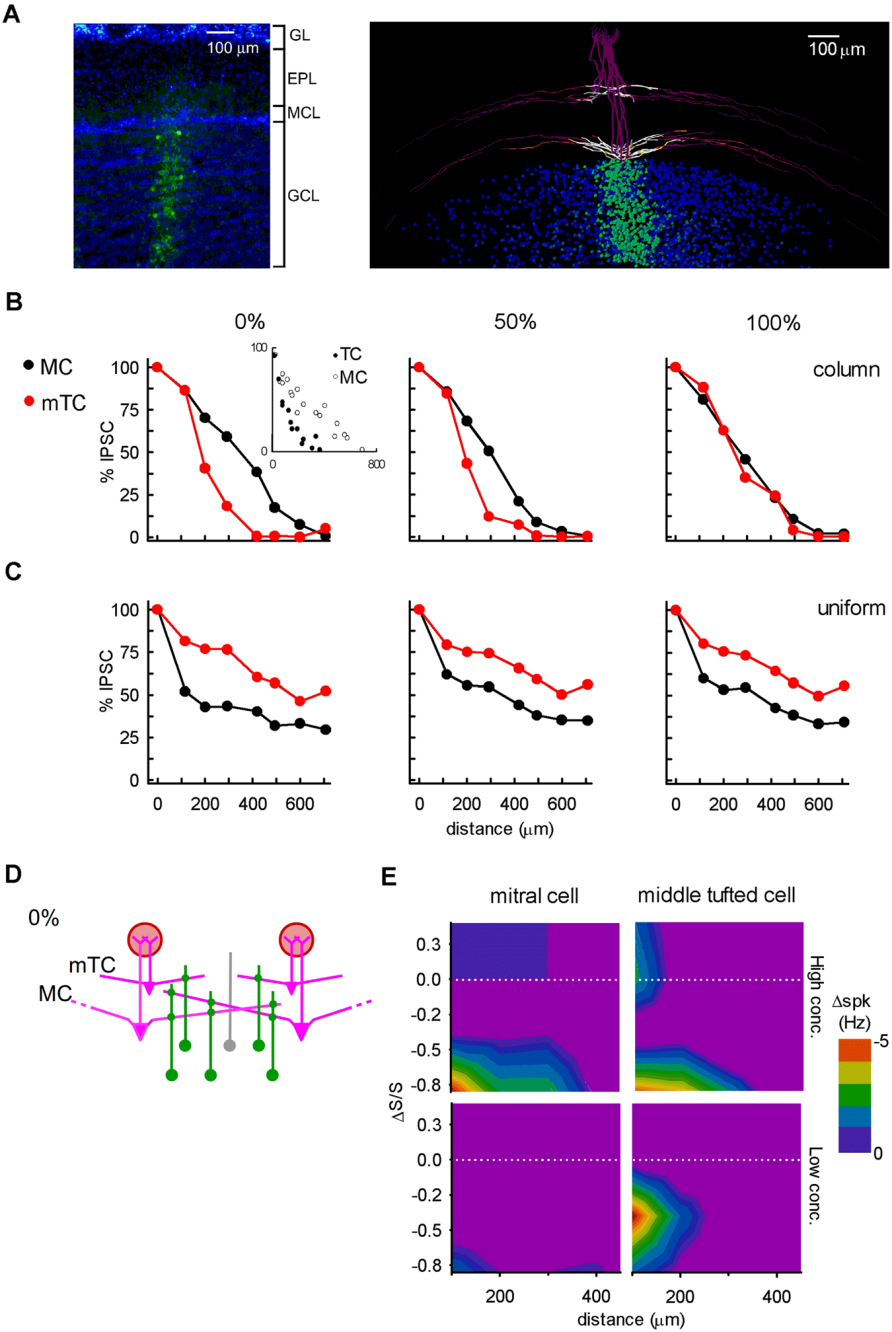
Spread of lateral inhibition in mitral and middle tufted cells through granule cell columns. (**A**) The GC column observed by experiment (left) (35), and reproduced by our model with realistic synthetic MCs and mTCs (right). (**B**) The spatial extent of the lateral inhibition in MCs (n = 5; black curve) and mTCs (n = 10; red curve) with GC columns at different percentages of GCs connecting with both MCs and mTCs, compared with the experimental results (inset). (**C**) Same as in (**B**) with uniform synaptic weights. (**D**) The different lateral extents of MC and mTC dendrites lead to different spreads of lateral inhibition depicted in B (0%). (**E**) Lateral inhibition causes a firing rate decrease in co-active glomeruli. The intensity is distance-dependent in both MCs (left) and mTCs (right), and depends on the relative strength of glomerular input (ΔS/S). This is more evident in mTCs even at lower odor concentrations (bottom graphs).

In summary, both our experiments and model suggest that MCs and mTCs have different susceptibilities to lateral inhibition. In particular, a new prediction from our model is that mTCs are quite sensitive to inhibition from only a few GCs, whereas MCs are relatively insensitive, although during activation of many GCs their firing rate can significantly decrease^13,25,26^. In contrast, GCs can readily make mTCs burst synchronously, if they share GCs through either a local microcircuit or when GCs receive synchronous excitation for example from cortical glutamatergic feedback. This mTC synchronization depends on the connections between GCs and the dendrites of the mTCs projecting to the involved glomeruli. There could be more than two glomeruli involved, as long as their connectivity follows the rule shown in the diagram (Fig. 3A,B, left panels). mTC synchronization also has a topographical feature in that the likelihood of the mTC lateral dendrites overlapping decreases with increasing distance^18,27–29^ (see also Fig. 4).

Experimental results obtained at room temperature^30,31^ suggested that synchronous inhibition could also trigger synchronous rebound bursts in MCs. However, recent experiments carried out at more physiological temperatures found that MCs exhibit significantly less bursting than mTCs^17^. Taken together, these previous results and our current results suggest that synchronous rebound bursts, mediated by a slow potassium current, can be robustly triggered in hetero-glomerular mTCs by synchronous inhibition conveyed via GCs. Modeling^32^ and experimental^33^ results suggest that MCs can also be synchronized by GCs under some conditions, for example, under highly correlated (i.e. similar) inputs. However, MC and mTC synchronization may rely on different time scales, with different overall effects on network dynamics.

### Connectivity of middle tufted cells within a glomerular unit

Experiments based on pseudorabies viral tracing^18,34^ revealed that the OB is organized in glomerular units (GUs) each formed by clusters or columns of neurons that are anatomically and functionally related to a single glomerulus (Fig. 4A, left). We have previously defined a GC column^13,27,35^ as that subset of GCs exhibiting potentiated reciprocal synapses with MCs and connecting the lateral dendrites of MCs or mTCs related to a GU^18,34^. As discussed in detail elsewhere^13,27,35^, the formation of a GC column consistent with those observed experimentally is an extremely robust process, which can be explained by considering the dynamical interaction between somatic action potentials backpropagating along the lateral dendrites and the consequent activity elicited in the reciprocal mitral–granule synapses. We have previously shown that as long as long-term depression and potentiation are induced by different levels of synaptic activity, a GC column will form below a strongly active glomerulus independently from the specific learning rule, and this process results in an overall lateral inhibition between GUs that decreases with distance^27^.

In Fig. 4A (right), we show the result of a simulation of a single strongly activated glomerulus in the current version of the model. To generate the GC column, we simulated odor learning as described in our previous works^12,13,27^. The somata of the GCs with potentiated synapses are labeled in green. As can be seen, the cluster formed a column similar to those seen in the experiments (Fig. 4, left). Going beyond the experiments, the model allowed us to visualize the sites of the potentiated synapses on the MC and mTC (see the white dendritic segments in Fig. 4A, right).

These columns robustly extended through the full depth of the granule cell layer (GCL), in qualitative agreement with experimental findings^34^, as shown by comparing the left panel with the right panel in Fig. 4A.

We next wished to determine how the columns reflect the connectivity between GCs and MCs and mTCs, but its experimental investigation is extremely difficult. It is known that the GCs are subdivided into at least two subtypes, superficial GCs and deep GCs^21,36–39^, and that they preferentially innervate the superficial and deep external plexiform layer^36–38^ where they connect to mTCs and MCs, respectively (*Methods*), along with a possible third type of GC that could connect to both^40^.

To obtain insight into the structure of the GC columns, we focused on the lateral inhibition between glomeruli as a function of distance. Christie and colleagues^28^ showed that the strength of lateral inhibition decreases with inter-glomerular distance (see Fig. 3 in ref.^28^, reported as an inset in Fig. 4B) with a wider spread between MCs than mTCs. Based on these results, in a series of simulations, we quantitatively characterized the lateral inhibition between pairs of glomeruli at different distances, assuming connectivity between GCs and MCs and/or mTCs. As shown by Christie and colleagues^28^, we measured the strength of the lateral inhibition in terms of the (normalized) somatic IPSC peak induced by stimulation of another GU. We found that the experimental findings could be reproduced only by assuming that GCs tend to form two distinct populations connected to either MCs or mTCs (0% co-connectivity of GCs with MCs and mTCs in Fig. 4B, left panel), given that columns are already formed. By increasing the number of GCs connected to both MCs and mTCs (Fig. 4B, middle panel), the difference in the extent of lateral inhibition between MCs and mTCs was decreased. When the overlapping connectivity was complete (Fig. 4B, 100%) there was no difference, in contrast with experiments^28^.

We also found that the organization of GC connectivity into columns was critical for reproducing the experimentally-observed patterns of lateral inhibition. This was tested by varying the distribution of potentiated synapses between GCs and MCs or mTCs, leading to either columnar or uniformly distributed connectivity (Fig. 4C). Independent of the amount of overlapping connectivity of GCs with MCs and mTCs, the lateral inhibition observed decreased with distance in a way inconsistent with experimental findings^28^. For the following simulations we hence assumed as the control condition the configuration corresponding to the left panel of Fig. 4B. Note the different spread of lateral inhibition between MCs and mTCs^28^ (Fig. 4B, left panel), when they have a well-formed GC column. A prediction of the model is that this is caused by the shorter average lengths of the lateral dendrites of mTCs compared with MCs^37,38,41^, resulting in a smaller lateral convergence on GC columns. This is illustrated in Fig. 4D. The shorter dendrites of the mTCs will eventually lose the inhibitory contribution from their GCs relative to MCs with distance from the soma (see gray GC in Fig. 4D). The model’s suggestion that MC and mTC circuits are separated at the GC-EPL level implies that each circuit contains a different degree of lateral convergence. Our results thus complement the more general experimental evidence of sparse and segregated GU connectivity^18,34^, and further suggest an even more segregated spatial convergence within the mTC circuit.

Finally, we wished to understand how these types of connectivity could lead to differences between MC and mTC responses as a function of odor concentration. For this purpose, we analyzed the firing rates of mTCs and MCs, using the circuit consistent with experimental findings and model predictions (Fig. 4E). A significant firing rate decrease is the typical consequence of co-active glomeruli^33,42^. This is mainly due to lateral inhibition via GCs^37^. Accordingly, we found a significant firing rate decrease in response to odor stimulation as a function of interglomerular distance for both MCs and mTCs. This is shown in Fig. 4E, where we plot the decrease in firing frequency as a function of interglomerular distance and the difference between the glomerular activation (ΔS/S, see *Methods*). In general, the firing rate decreased more strongly when the glomerulus was less activated (negative values of ΔS/S, below the white lines). MCs and mTCs responded in different ways to different odor concentrations, in both intrinsic firing activity and effectiveness of lateral inhibition via GCs. In agreement with *in vivo* experimental evidence^43^, mTCs are generally more responsive than MCs especially at low odor concentrations. At higher concentrations, firing rate was increasingly inhibited in both MCs and mTCs; by comparison, at low concentrations, an appreciable firing rate decrease was observed only in mTCs, with inter-glomerular distance up to 200 μm (top). It is well established that mTCs are more sensitive to lower odor concentrations than MCs, presumably reflecting their higher input resistance^17^.

Taken together, these results suggest that MCs and mTCs form two parallel pathways connected to separate subsets of GCs. Lateral inhibition could thus spread through GCs only in a MC-MC or mTC-mTC way, but not MC-mTC.

### Connectivity of middle tufted cells with the glomerular circuitry

**Figure 5:**
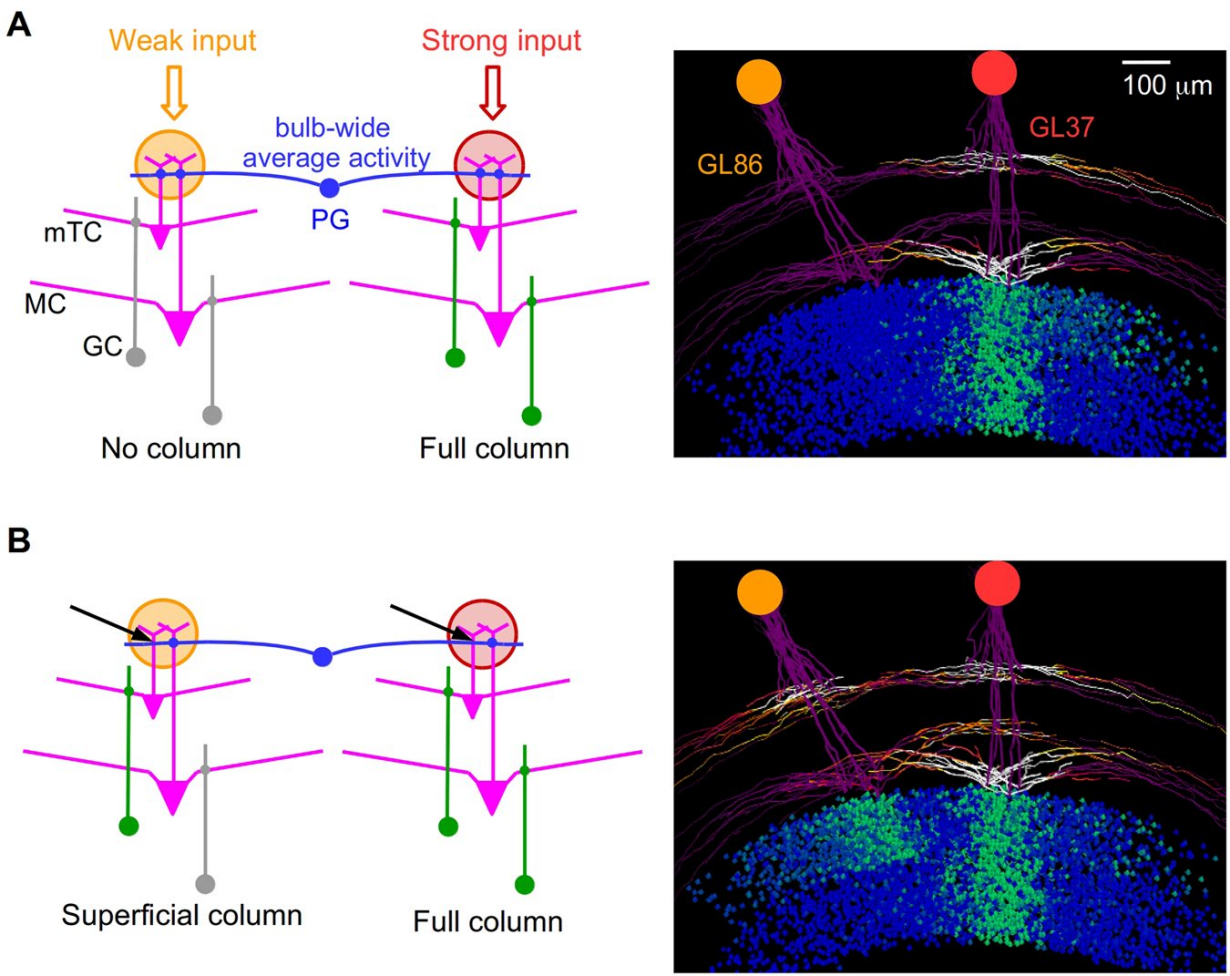
Mitral and middle tufted cells are connected by a similar juxtaglomerular circuitry. (**A**) The glomerular layer turns off every glomerulus that receives a weak input (orange, left). Only a strong input (pink, right) can form a GC column (right), which extends through the full depth of the GCL. (**B**) Same as in (A), but with mTCs disconnected from the glomerular circuitry (left); black arrows indicate the missing connections between PGCs and mTCs; this leads to the formation of a GC column limited to the superficial location of the mTCs. GL86 and GL37 are specific glomeruli in the synthetic neuron circuit.

In the previous section, we focused on GU interactions at the external plexiform-GC layer. We now consider the interactions between MCs and mTCs at the glomerular layer (GL), to explore the influence of intraglomerular connectivity. The overall circuit was described by Cavarretta et colleagues^13^ and is depicted in Fig. 5A, left. The glomerular circuitry is based on a model^44–46^ where the glomerular layer mediates inhibition onto the tuft dendrites of both MCs and mTCs, as supported by experimental evidence^47^. As a result, glomerular layer inhibition, mediated by PGCs, suppresses the activity in MCs and mTCs that receive weak input.

As described above, a GC was connected to either MCs or mTCs. The two glomeruli received weak or strong odor input (yellow and red, respectively). A strongly activated glomerulus results in a GC column, according to the process described in the previous section (see also *Methods* for more details). The inhibition mediated by the glomerular layer suppresses activation of both mTCs and MCs in the weakly stimulated glomerulus (left). Under these conditions, learning would result in a GC column only below the strongly activated glomerulus (Fig. 5A, right), extending across most of the GCL, consistent with experiments in which 95% of the GC columns extended through the full GCL depth^18,34^. However, if we assume that, in the glomerular layer, the PGCs make contact with only a single cell type (for example only with MC tufts, Fig. 5B, see arrow), there is no feedfoward inhibition from the strongly activated glomerulus of the mTC in the weakly activated glomerulus, but there is feedforward inhibition of the MC. This would produce a short partial column that is not seen experimentally. The model thus predicts that glomerular layer circuitry acts in the same (or very similar) way on both MCs and mTCs.

This result has important functional implications. The glomerular layer mediates bulb-wide average activity and contrast enhancement, realizing a “relational odor representation” that is concentration invariant^44–46^. It also leads to sparse glomerular activation^13^, even with dense glomerular input, which in turn is reflected in the sparseness of the GC columns^13,18,34^. Taken together, these results suggest that both MCs and mTCs must be controlled in a similar way by the glomerular circuitry.

### Deep short-axon cell inhibition may coordinate olfactory bulb output: mitral cells

The results indicate two distinct processing pathways, and raise the question of how they are coordinated in sending the output of the OB to the olfactory cortex. Two properties are usually considered key for odor coding: cell firing rate and/or synchronization. We hypothesized coordination would likely involve actions of at least one of several possible inhibitory interneurons at the level of OB output. The best understood of these interneurons is the dSAC. The dSACs are a population of inhibitory neurons located deep to the MC bodies in the superficial GCL. Experimental findings have suggested that: *i*) only mTCs significantly innervate the GCL^16^, *ii*) the principal neurons (MC and mTC) do not significantly excite GCs through their axons^48^ but through their dendrites, *iii*) dSACs are recruited by multiglomerular activation^49^, *iv)* dSACs respond to current injection with persistent high frequency firing, somewhat independently from the stimulation protocol^50–52^, and *v)* their activation induces a tonic inhibition on GCs strong enough to block action potentials^19,53^.

**Figure 6:**
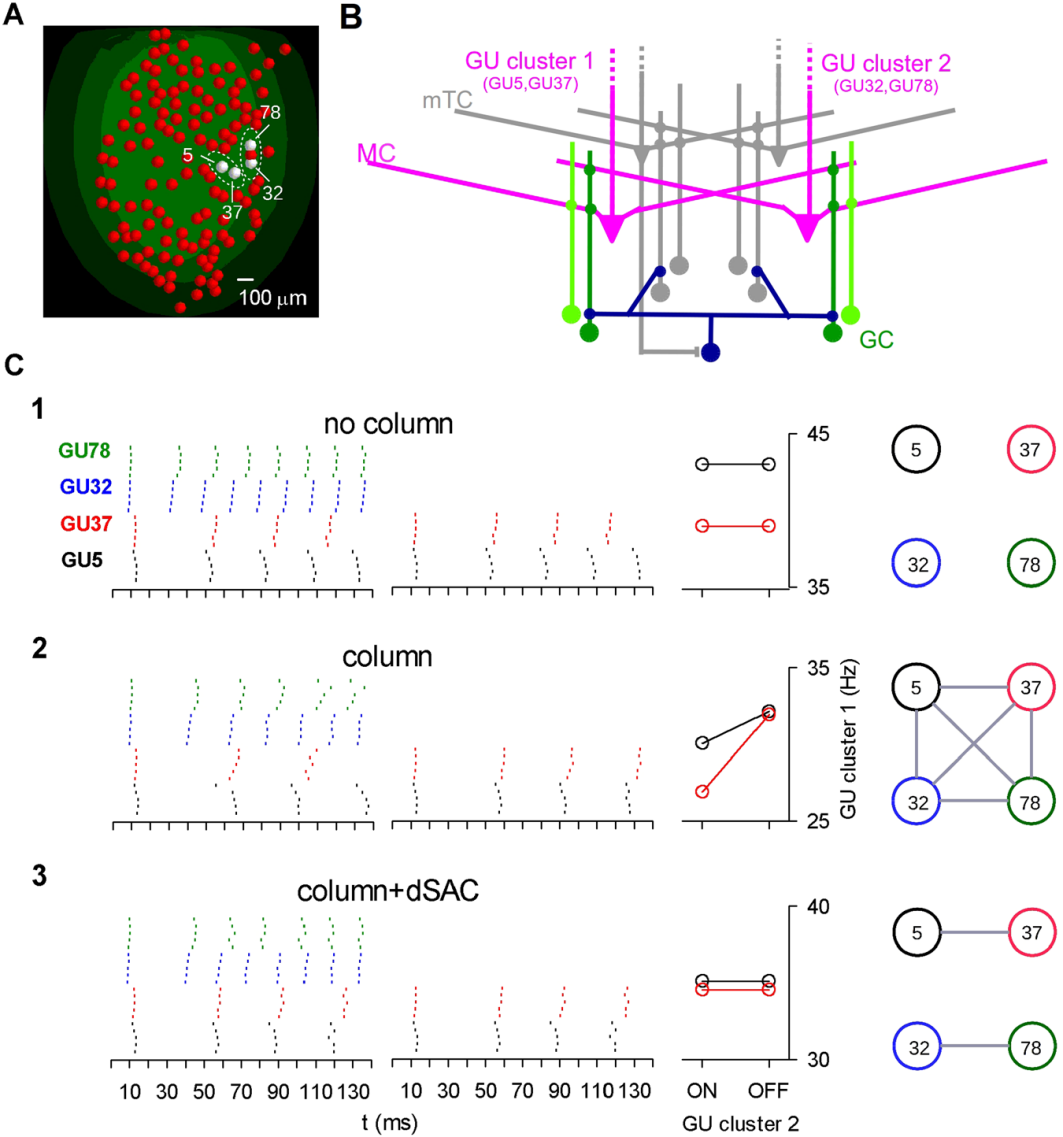
Modulation of mitral cell firing by deep short-axon cells in different glomerular unit clusters. (**A**) Shows a subset of glomerular units (GU) organized in two clusters (GU 5, 37 and GU 32, 78). (**B**) The GU clusters 1 and 2 and their proposed connectivity with dSACs. Note that the dSAC (blue cell) selectively targets GCs (green cells) connected to different clusters. (**C**) Raster plots for the MCs in the four GUs (*left*), firing rates for GUs in cluster 1 when cluster 2 is active (ON) or silent (OFF) (*middle*), and connectivity diagram with links representing interacting glomeruli (*right*). Three configurations are shown: (C1) without GC columns, no connectivity between GUs; (C2) with GC columns, connectivity is all-to-all; (C3) after adding dSACs, connectivity is established for GUs within the same cluster.

These findings, and the available experimental evidence^16,19,48–53^, suggested that dSACs are selectively activated by mTCs but not MCs^16,28,49^. We therefore tested several different connectivity rules between dSACs and GCs. We hypothesized that previous odor learning resulted in the dSAC targeting (and thus inhibiting) GCs connecting GUs belonging to different clusters. To test the functional consequences of this circuit, we used the set of simulations illustrated in Fig. 6. We selected two GU clusters (GU5–37 and GU32–78) as indicated in Fig. 6A. These GUs represented four real GUs in the dorsal part of the OB (see Fig. 2 in ref.^54^). The proposed circuit is schematically represented in Fig. 6B, including a dSAC (blue cell), MCs representing all those belonging to a given GU (magenta cells), and GCs connecting to MCs belonging to GUs of the same group (light green cells) or to MCs belonging to different GU clusters (dark green cells).

**Figure 7:**
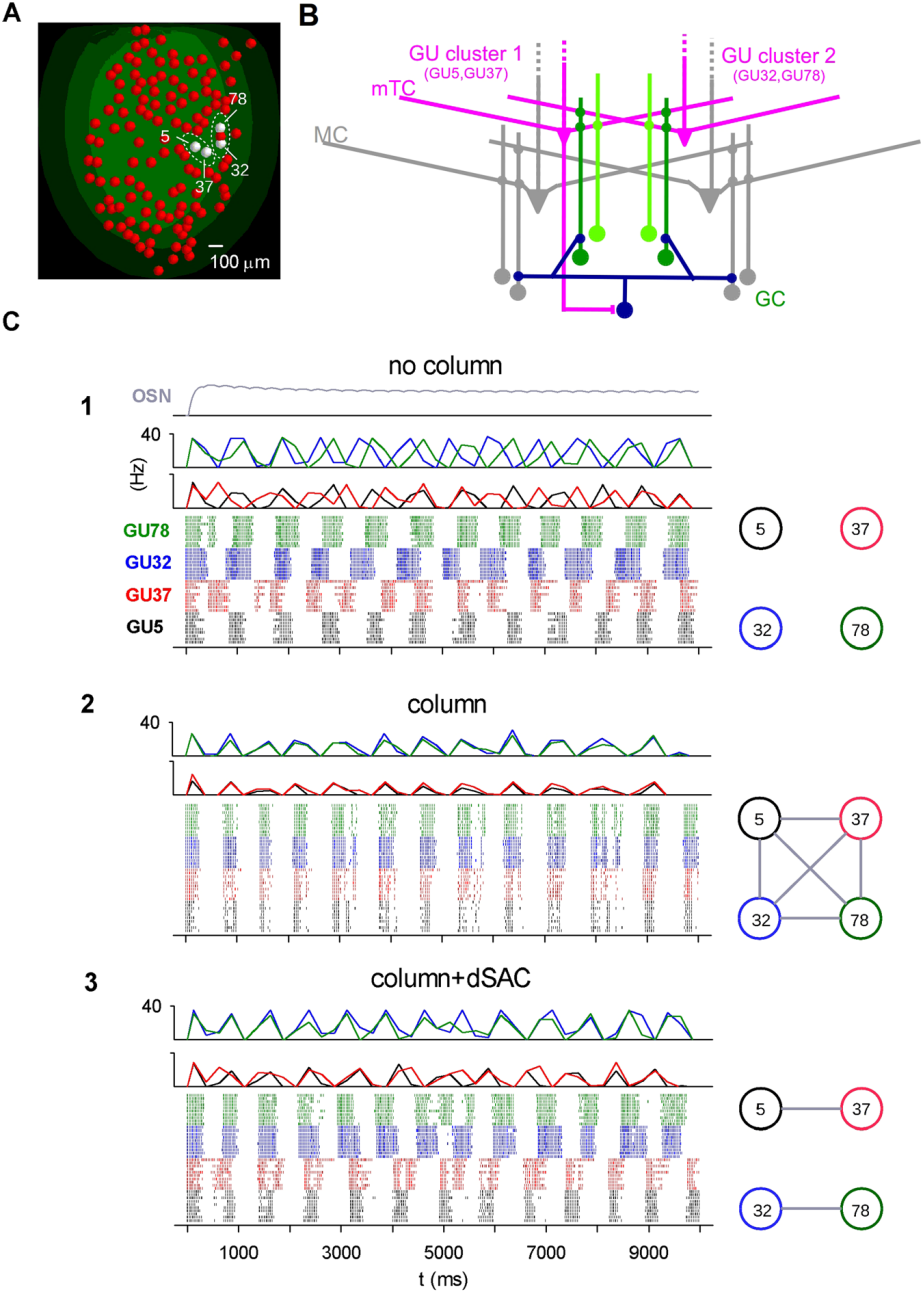
Deep short-axon cells organize synchronization among middle tufted cells in different glomerular unit clusters. (**A**) Same configuration as in Fig. 6A for mTCs. (**B**) Same configuration as in Fig. 6B, except that the mTCs are highlighted in purple and their connected GCs in green. (**C**) Raster plot of mTCs in four glomerular units, with OSN activation (grey curve), and the related post-synaptic stimulus histograms (top) under the same three conditions as in Fig. 6: (C1) without GC columns, (C2) with GC columns, and (C3) with GC columns and dSACs; *right*: connectivity diagrams illustrating the interacting glomeruli for each case.

We ran simulations to test MC firing in response to an odor input under these different connectivities. We followed the experimental protocol used in previous reports^33,37^, to test when a GU was activated alone or with another GU. In Fig. 6C, the raster plots of the MCs belonging to each GU are shown under the different conditions of: (1) no GC columns, (2) with GC columns, and (3) with GC columns and dSACs. The effects of these network configurations were tested by running two independent simulations activating both (ON) or only one (OFF) GU cluster. From the observed firing rate (middle graphs), one could predict whether the GUs were functionally connected, as indicated by a link in the connectivity diagrams (Fig. 6C, right), illustrating the network configuration in each case. Note that stimulation of a GU activated both MCs and mTCs; we show their action separately in Figs 6 and 7 to highlight the selective effect of mTCs on dSACs.

For the case of MCs without columns (Fig. 6C1), activity of GUs in cluster 1 was not affected by activity in cluster 2 (Fig. 6C1, red and black raster plots). Note that to reflect physiological variability the various GUs did not receive exactly the same input. Under these conditions, the cells connected to the GU in cluster 1 maintained their firing rate independently whether cluster 2 was ON or OFF (see middle graph: the GUs were effectively disconnected from each other (see diagram on the right)).

If each GU had a GC column (Fig. 6C2), the firing rate in cluster 1 changed when cluster 2 was either ON or OFF (graph). In particular, because of the effect of their own columns, the GUs in cluster 1 tended to synchronize when cluster 2 was OFF (Fig. 6C2, middle plot), whereas this could not happen with cluster 2 ON. This configuration can be represented as an all-to-all GU connectivity in the diagram (Fig. 6C2, right), since each GU could affect the firing rate of all the others.

Finally, the effect of dSACs is shown in Fig. 6C3. The raster plot shows that MCs belonging to a given cluster could now maintain their synchronization and firing rate independently of the activity in the other cluster. This effect was most evident in the same average firing rate for GU cluster 1 independently of cluster 2 being ON or OFF (see graph); it was caused by the inhibitory dSACs action on GCs connected to different clusters; this effectively disconnected the two clusters (Fig. 6C3, diagram on the right). These results suggest that dSACs could have an important role in promoting a network configuration that allows dynamic separation of activity patterns between clusters but not within the same cluster. We thus hypothesize that the spatial decorrelation over time promoted by the MC circuit^13^ may be limited to clusters of GUs rather than the entire OB.

### Deep short-axon cell inhibition may segment spatial odor representation: effect on middle tufted cells

We finally turn to the effect of dSACs on mTCs (Fig. 7), by considering the same GUs (Fig. 7A) connected as above (Fig. 7B). As shown in Fig. 3, mTCs differ from MCs in showing synchronous bursting following a common GC input. Figure 7C shows how the same connectivity configuration as in Fig. 6C influenced mTC synchronization of bursts evoked by a relatively low intensity input, i.e. in a range within which mTCs would exhibit bursts. For these simulations, we used a 3 Hz respiration cycle. At this rate, the level of OSN activation was almost constant, as observed experimentally^54^, and in our model (Fig. 7C1, grey trace). This relatively constant input triggered bursting responses because of the intrinsic membrane properties of mTCs (see Fig. 3), as also shown experimentally^17^. Without columns (Fig. 7C1), mTCs in the different GUs fired bursts at independent intervals (Fig. 7C1, raster plots), reflecting the lack of connectivity (Fig. 7C1, diagram). In the presence of columns, synchronization occurred between all GUs (Fig. 7C2, raster plots), corresponding to an all-to-all connectivity (Fig. 7C2, diagram). This strong synchronization was promoted by the specific electrophysiological properties of mTCs (Fig. 3). The presence of dSACs (Fig. 7C3) generated synchronization within, but not between, clusters (Fig. 7C3, raster plots), reflecting a corresponding connectivity within, but not between, them (Fig. 7C3, diagram). More quantitative results are summarized in Supplementary Table S1. These results therefore suggest that dSACs could act as cluster separators on the output to the olfactory cortex (see *Discussion*).

## Discussion

These results introduce the basic mechanisms and features of OB circuits that contain mTCs, and provide a conceptual basis toward an explanation of their contribution to odor processing. This has led to three main advances toward a more comprehensive model of information processing in the OB.

### Two parallel pathways and two layers of processing

The new model builds on experimental results to support the hypothesis that MC and mTCs function as two parallel output pathways. This is not immediately obvious at the glomerular level, where the two pathways begin by processing the olfactory input through common synaptic circuitry. The model suggests that the common processing at this level is a specific requirement to be consistent with experimental findings (Fig. 5). Functional separation begins in the subsequent processing within the deeper microcircuits of the GCs. The hypothesis of this functional separation is supported by several experimental findings. At the anatomical level, it is known that there are two populations of GCs, superficial and deep, related to mTC and MC lateral dendrites, respectively. In terms of connectivity, imaging of viral tracing through the lateral dendrites shows that GCs connect to either MCs or mTCs but rarely to both. The model suggests that this connectivity pattern is also a specific requirement to accommodate other experimental data (Fig. 4).

This separation takes on significance in relation to the physiological properties of the MC and mTCs. Their firing properties: MCs tend to give regular firing action potentials in response to injected current whereas mTCs show bursting at lower levels of current injection. Our experiments also showed that they give rebound bursts in response to hyperpolarizing current pulses. In addition, the mTCs have a higher sensitivity to stimulation than the MCs in the model, as in experiments. This correlates with the higher input resistance of the smaller mTCs, as well as with the actions of the GCs (Fig. 4E). All these properties have been combined in our model so that the mTCs respond to odor with bursting and a tendency for higher sensitivity to odor stimulation, whereas MCs respond with regular spiking. The consequences for odor coding are discussed next.

### Odors are encoded by glomerular units and cell columns

Experiments showed that natural odors excite large ensembles of glomeruli that are highly overlapping^5^, while theoretical studies showed that the glomerular circuit may disambiguate these odor inputs, implementing a winners-take-all mechanism that leaves a sparse ensemble of active glomeruli interspersed in a large population of suppressed glomeruli^13,44–46^. As a consequence, the overlap between ensembles of responsive glomeruli is strongly reduced. We have previously suggested^13^ that this transformation of the sensory inputs is crucial for the sparse formation of GC columns observed experimentally^34^. Here we further highlight the functional relevance of the columns, addressing their possible interaction with the mTCs. These structures may be the key players for interglomerular interaction. The model predicts that the function of a GC column in this case is to modulate the lateral inhibition between populations of cells belonging to different glomeruli (Fig. 4), populations that we have termed “glomerular units (GUs)”. We have finally suggested that lateral inhibition may lead to a fast synchronization between mTCs belonging to different GUs (Fig. 7).

### Coordination of the parallel pathways by dSAC

It is plausible to assume that all sparse glomerular units activated by a given odor should be interconnected in providing input to the OC. As noted in the *Results* and in our previous work^13,27^, glomerular units may interact via GC columns. In addition, here we propose that their interactions may be coordinated by dSACs. This coordination may organize the glomerular units into larger odor-dependent entities termed “glomerular unit clusters”.

How do dSACs coordinate the two MC and mTC pathways? Our results point to the organization of the dSAC circuit. On the output side, experimental data shows that dSACs can inhibit GCs^49,50,53^. On the input side, direct experimental evidence is lacking, though several experimental clues support our hypothesis that mTCs are responsible for dSAC activation^49,52^. Additionally, we have suggested that dSACs preferentially inhibit GCs connected to MC or mTCs belonging to different GU clusters. Under these conditions, our model predicts that the MCs belonging to the same GU cluster can be interrelated through their similar average firing rates (Fig. 6), whereas the unifying property for mTCs is the tendency to burst synchronously (Fig. 7C).

### Connectivity between the two parallel pathways and the olfactory cortex

Both MCs and mTCs project to OC. The biggest subregion of the OC is the piriform cortex (PC), which is subdivided into anterior (aPC) and posterior (pPC) areas. MC axons innervate the entire PC but the aPC is the main axonal target^16^, whereas associative fibers arising within the cortex are the main input for pPC^55^. By comparison, mTCs project substantially as well to the anterior olfactory nucleus (AON) and olfactory tubercle (OT). MC and mTC axons spread in the most superficial layer of the OC, where they make excitatory synapses with distal apical dendrites of pyramidal cells and feedforward inhibitory neurons^55^. All these axonal projections to the OC appear heavily intermingled, lacking the columnar organization of the OB^18^.

The different subregions of the OC are directly or indirectly involved in many cognitive functions: the OC mediates higher level cognitive functions^56,57^; the AON seems to be involved in a wide variety of other different functions^58^; the OT encodes reward-driven and anticipatory responses^9,59^, with dopamine modulating OT activity^60^ and optimizing the adaption of response to reward shift^61^. Accordingly, although the different subregions of the OC are interconnected, it has been experimentally suggested that they encode different aspects of odors, reflecting the different activities of MCs and mTCs^59^. All these observations suggest a general picture in which the parallel odor coding performed in the OB carries over into those cortical areas that are primary targets of MCs and mTCs.

### Using the model to test hypotheses regarding mitral-middle tufted cell odor processing

With this level of realistic detail, the model can be used to test hypotheses regarding the differential activation of the two parallel pathways. The model can serve a useful purpose in directing future experiments to testing our hypothesis that dSACs preferentially make synapses with GCs connected to MCs or mTCs belonging to different GU clusters. Current research indicates that various subclasses of dSACs, as well as other interneuron classes, may contribute to this role, or to other functions within the OB^37,52^. Another possibility brought to our attention is a subclass of olfactory receptors^62^ that could be related to differential activation of mTCs compared with MCs in the glomeruli, leading to distinct functional roles in odor decoding via feedforward inhibition for inducing input synchrony between multiple cognate olfactory receptors in the OC as the observed information redundancy reduction from stimulus-experience dependency to odor-quality dependency^63^.

### Computation in the olfactory bulb

The realism of our model allows us to analyze within the network framework the interactions between single neurons, in order to understand the computations carried out in the OB in the context of odor discrimination.

As shown in Table 1, the mTC firing correlation between clusters is lower with GC columns and dSACs than without GC columns, even though there was no interaction between clusters in either case. This is a typical function performed by clustering algorithms. A desirable property of these algorithms is indeed the ability to strengthen the similarity between patterns within clusters, while the difference between patterns in different clusters is enhanced (e.g. the UPGMA algorithm)^63^. Similarly, the segmentation in spatial regions (which are conceptually equivalent to glomerular clusters) encoding different features is used for visual or word recognition^64,65^, where regions are outlined by edge detection; this computation is equivalent to that performed by dSACs.

**Table 1:** Cross-correlation within and between clusters under different configurations.

		No column				Columns				Columns + dSACs			
		Cluster 1		Cluster 2		C**luster** 1		Cluster 2		Cluster 1		Cluster 2	
	GL	5	37	32	78	5	37	32	78	5	37	32	78
Cl. 1	5	1	0.05	0	0.72	1	0.94	0.92	0.91	1	0.71	−0.02	−0.12
	37	0.05	1	0.03	−0.05	0.94	1	0.93	0.93	0.71	1	−0.02	−0.17
Cl. 2	32	0	0.03	1	0.10	0.92	0.93	1	0.96	−0.02	−0.02	1	0.83
	78	0.72	−0.05	0.1	1	0.91	0.93	0.96	1	−0.12	−0.17	0.83	1

Furthermore, theoretical^66^ and experimental^6–9^ findings suggested that the OC works as an associative memory. In this scenario, our model suggests a possible way in which the GU clustering performed by the dSAC circuit can organize the OB output to facilitate associative memory operations, as suggested by previous theoretical studies^66^.

## Methods

### Computational methods

All simulations were carried out with a fully integrated NEURON+Python parallel environment (NEURON v7.4) on a BlueGene/Q IBM supercomputer (CINECA, Bologna, Italy). The model and simulation files used for this work are available for public download under the ModelDB section of the Senselab database suite. The computation and implementation details of the full 3D model of the OB have been described in detail in our previous papers^11–13^. Here we report only those related to the implementation of the mTCs. The full system used in this work included 635 MCs, 1,270 mTCs, and 191,485 GCs.

The connectivity between mTCs and GCs was generated by the same random algorithm used for MCs, and described in previous works^13,27^. The same density of reciprocal synapses was used for MC (0.1 syn/μm)^11–13^ and mTC (0.1 syn/μm)^13,27^ lateral dendrites. Both MCs and mTCs received the same type of glomerular input^13^.

### Synaptic plasticity and granule cell column formation

Synaptic plasticity was implemented for the dendrodendritic synapses connecting GCs with MCs or mTCs, using the same plasticity rule explained in several previous papers^12,13,27,35^. Briefly, all synaptic weights started at zero and, in response to an odor input, each component (inhibitory or excitatory) of each dendrodendritic synapse was independently modified according to the local spiking activity in the lateral dendrite of the MC/mTC or in the GC synapse. As described in detail elsewhere^12,13,27,35^, the formation of synaptic clusters consistent with those observed experimentally is a robust process. It can be understood by considering the following dynamics: *a)* a strong odor input causes MC/mTCs to fire at a high-frequency; *b)* somatic APs backpropagate along the lateral dendrites and potentiate excitatory synapses along their way, activating GCs; *c)* GCs begin to fire at a high-frequency, potentiating inhibitory synapses on the lateral dendrites of MCs and mTCs, *d)* inhibition from GCs hinders AP back-propagation far from the soma, thus reducing, locally, the dendritic firing frequency, and *e)* this finally results in the selective depression of synapses far from the soma of the active MC/mTC. Therefore, as long as: *1)* action potentials backpropagate along the lateral dendrites, *2)* GCs form dendrodendritic connections, and *3)* long-term depression and potentiation are induced by different levels of synaptic activity. A GC column will be thus formed independently of the specific learning rule.

### Odor inputs

The glomerular excitation generated by an odor input and conveyed to MCs and mTCs was represented by dose-response curves expressed as Hill functions, with a spatial distribution and a time course based on experimental evidence^13^.

### Glomerular layer circuits

For this component of the model, we have used the same approach as in our previous work^13^, extended to include mTCs. The overall circuit is based on that suggested by Cleland and Linster^44–46^, where the intra- and inter-glomerular circuitry were implemented through a set of empirical equations describing the overall effects of the glomerular layer circuitry, implicitly taking into account the effects of PGCs and ETCs. The glomerular activation (*S*, Fig. 4) was calculated as the overall response (in terms of firing rate) of the OSNs converging on a GU (eq. 2 in ref.^13^). For complete details, see ref.^13^.

### Membrane properties of mitral and middle tufted cell models

MCs and mTCs have strong anatomical similarities; morphologically, a mTC resembles a scaled down MC^40^. Both cell types are excited by OSNs^40,67,68^ and contribute to the columnar organization of the OB^18,34^. However, electrophysiological recordings in mice have revealed significant differences between MCs and mTCs^17^, due to different intrinsic membrane properties^17^. These differences in turn lead to different responses and properties of lateral inhibition^28,69^, especially when induced through GCs^37^.

The membrane properties were chosen to reproduce specific experimental findings. In the slice, a significant amount of lateral dendrites was likely amputated. In order to account for this, to estimate model parameters we used a reduced morphology including only two lateral dendrites for a MC and mTC. The cell body was approximated by a sphere of 20 μm diameter. The apical dendrite was 4 μm in diameter and 400 and 250 μm in length for MCs and mTCs, respectively; it branched into four small tuft dendrites, each 0.8 μm in diameter and 80 μm in length. The lateral dendrites were 3 μm in diameter and 800 μm in length in MCs, and 2.5 μm in diameter and 600 μm in length in mTCs. All these values are consistent with previous experimental findings^40^. The morphology of the axon was the same as used in previous reports^69,70^.

Passive properties are summarized in Supplementary Table S1. All membrane properties were uniform in all compartments, except the axon. The passive properties were the same for MCs and mTCs (Supplementary Table S1), in agreement with experimental measurements^17^; we preserved the ratio between the passive properties of the axonal sections with the others, as shown in previous studies^69,70^. With these parameters, the cell morphology yielded an input resistance of 91.5 and 125.3 MΩ for MCs and mTCs, respectively, similar to experimental measurements (94.3 +/− 40.5 and 111.8 +/− 51.6 MΩ, respectively)^17^.

With regard to active properties (Supplementary Table S2), for MCs we used Na, K_A_, and K_DR_ –type conductances, with the same peak conductance as in our previous work^11–13^. For the mTCs, we found that by including a slow K conductance (K_S_)^71^, instead of the K_DR_, we were able to obtain a good qualitative agreement with the observed mTC burst^17^. Only K_A_ and Na were present in the axon, with a Na density 30-fold higher than in the other compartments, in order to reproduce the experimental observation that the action potential initiates close to the soma with weak current injections^17^.

In summary, in comparison with previous models^11–13^, the MC model was extended by adding an axon and using updated values of passive membrane properties, whereas the mTC model, adapted from the MC model (see below), was new. We have chosen to update the MC model because it takes into account more recent experimental findings^17^, and allows for a more direct comparison between MCs and mTCs.

Specific membrane properties were matched to the specific experiments in which they were obtained from slice preparations; these values were then used to extrapolate to values for the full models in the simulations. Populations of synthetic morphologies were obtained with a custom algorithm^11–13^. The models predicted that the input resistances of MCs and mTCs with full dendritic trees would be 27.4 +/− 0.8 and 65.2 +/− 2.7 MΩ, respectively. The lower values reflected the larger conductance loads of the membrane in the presence of a full dendritic tree.

### Morphological properties of middle tufted cells

Starting from the experimental full 3D mTC morphologies (n = 6), a population of synthetic cells was generated with the same algorithm used for MCs^11–13^. The process was based on the distribution of dendritic path-length, branch length, and branching probability (Fig. S2). To test the validity of the synthetic mTC morphologies, we compared the Sholl plots and the proportion of dendrites vs branch order against the experimental distributions; they were statistically indistinguishable (Wilcoxon Signed Rank, test p = 0.949 and p = 1.0, respectively; Fig. S2). In mTCs, as well as in MCs, the total length of lateral dendrites determined the lateral extent of its interactions with GCs. The distribution for our model cells peaks at approximately 8,500 microns (Fig. S2). This length is in turn correlated with the input resistance.

### The granule cell model

GC morphology was the same as used previously^11–13^ with a few minor adjustments for passive (Supplementary Table S1) and active (Supplementary Table S2) properties. We implemented two GC subtypes: superficial (sGC), related to the more superficially located mTCs, and deep (dGC), related to the deeper MCs. A dGC has a higher K_DR_ and lower Na conductance than a sGC, to take into account the experimental finding that it is less excitable^49^. Overall, in the models for sGCs and dGCs, the relation between firing rate and current intensity was consistent with experimental findings^49^ (not shown). The average value of the input resistance was 603.2 +/− 36.3 MΩ, in agreement with experimental measurements^49^.

### The deep short-axon cell model

For a specific set of simulations, we implemented a dSAC using a ball and stick model with a 1,400 μm length dendrite, consistent with *in vitro* experiments^49^. The intrinsic membrane properties (Supplementary Table S1) where chosen to obtain an input resistance of 278.3 MΩ, close to experimental measurements^18^. In the simulations, we used dSACs with a doubled dendritic length to take empirically into account the membrane lost in the slice. In the absence of specific experimental evidence, we used the same active properties as for GCs. The dSACs were excited by mTC axons, which release glutamate and trigger NMDA/AMPA receptor activation^72^. To take into account this mechanism, we used double-exponential synapses with a rise and decay time constant of 2 and 200 ms, respectively. These properties were sufficient to reproduce the persistent firing, somewhat independently of stimulus strength^50^. The peak synaptic conductances between dSACs and GCs were set to be strong enough to completely shut down the GCs during strong odor inputs. We did not explore in detail the parameter space, which depended on many factors that were not considered relevant.

### Experimental reconstruction of middle tufted cells

We used full 3D reconstructions of six mTCs (Fig. 1B) reported in previous work^16^. Briefly, spiking activity of mTCs was recorded using juxtacellular recording from anesthetized adult C57BL/6 mice. mTCs responded either to 2, 34, 5-trimethylthiazoline (TMT) and 2-methylbutyric acid (2MBA), which were electroporated with biotinylated dextran amine (BDA). After 3-day survival, mouse brains were fixed, sectioned and processed with avidin-biotin-peroxidase complex (ABC) for BDA visualization. Dendrites and axons of mTCs were three-dimensionally reconstructed from digital data using Neurolucida.

### Electrophysiology

Whole-cell current-clamp recordings were made from mTCs in acute horizontal slices (310 μm thick) prepared from ~4 w-old C57BL/6 mice of both sexes as previously described^53^. Action potential firing was evoked on interleaved trials with equal-amplitude (157.1 ± 53.5 pA, n = 7) constant step current injection, and constant step current injection with brief pauses in depolarization (50 ms-long pauses at 4 Hz). Spike times were calculated using a 20 mV/ms action potential-detection threshold. Spike-time reliability was calculated as previously described^73^. All experiments were completed in compliance with the guidelines established by the Institutional Animal Care and Use Committee of Carnegie Mellon University and the University of Pittsburgh.

## Electronic supplementary material

Supplementary Information
