# Supplementary material for: Parallel odor processing by mitral and middle tufted cells in the olfactory bulb
Source: Sci Rep. 2018 May 16;8:7625. doi: 10.1038/s41598-018-25740-x (PMC5955882; doi:10.1038/s41598-018-25740-x)
Supplement: Supplementary file 1 — Supplementary Information [file 41598_2018_25740_MOESM1_ESM.pdf]

Parallel odor processing by mitral and middle tufted cells in the olfactory bulb

Francesco Cavarretta[1,2], Shawn D. Burton[3,4,5], Kei M. Igarashi[6], Gordon M. Shepherd[1], Michael L. Hines[1], Michele Migliore[1, 7*]

[1] Department of Neuroscience, Yale University School of Medicine, 06510, New Haven, CT

[2] Dipartimento di Matematica "Federigo Enriques", Universita' degli Studi di Milano, 20133, Milano, Italy

[3] Department of Biological Sciences, Carnegie Mellon University, 15213, Pittsburgh, PA

[4] Center for the Neural Basis of Cognition, University of Pittsburgh, 15213, Pittsburgh, PA

[5] Department of Neurobiology and Anatomy, University of Utah, 84112, Salt Lake City, UT

[6] Department of Anatomy and Neurobiology, and Center for the Neurobiology of Learning and Memory, University of California, 92697, Irvine, CA

[7] Institute of Biophysics, National Research Council, 90146, Palermo, Italy

The different membrane properties of mitral and middle tufted cells

To implement MCs and mTCs model cells, our first experimental constraint on the circuit model was their response to somatic current injection. Typical examples of a model MC and mTC are shown below, in Fig. S1A. Their responses to somatic current injections reflected the different input resistance and active membrane properties[17] (see *Methods*), and resulted in a higher firing rate-vs-current intensity (F-I) for mTCs (Fig. S1B). The experimental data (see Fig. 4E in ref. 17[17]) gave a smaller difference, which may be caused by the recordings being made in slice preparations which cause partial amputation of the dendritic tree. The firing patterns were also distinctly different with increasing current (Fig. S1C). MCs showed a gradual increase in tonic firing, whereas mTCs showed characteristic high frequency bursting at low intensities, changing to high frequency tonic firing at high intensities, consistent with experiment[17]. The response latencies (not shown) were similar (1-2 ms) and relatively invariant with the current intensity, consistent with experimental findings in the presence of GABA blockers[18].

The functional consequences of the different intrinsic properties were analyzed with a stimulation protocol including hyperpolarizing pulses (Fig. S1D). Each hyperpolarizing pulse resulted in a robust rebound burst only in the mTCs (Fig. S1D, right); in our model, the burst was mediated by the slow potassium channel (K_S). Supplementary Figure S1E shows the dynamics of the ionic channels underlying mTC burst generation. The brief hyperpolarizing pulse (indicated by the blue box) significantly altered Na^+ channel activation/inactivation (top) and K_S activation (middle, red curve), transiently reducing the influx of Na^+ and K^{+-} currents. After the current pulse, the K_S channels recovered more slowly than the Na channels (Fig. S1E, bottom, gray curve), leading to a Na^+ current that finally triggered a rebound burst. In summary, these results suggested the experimentally testable prediction that mTCs can robustly produce rebound spiking at physiological temperatures.

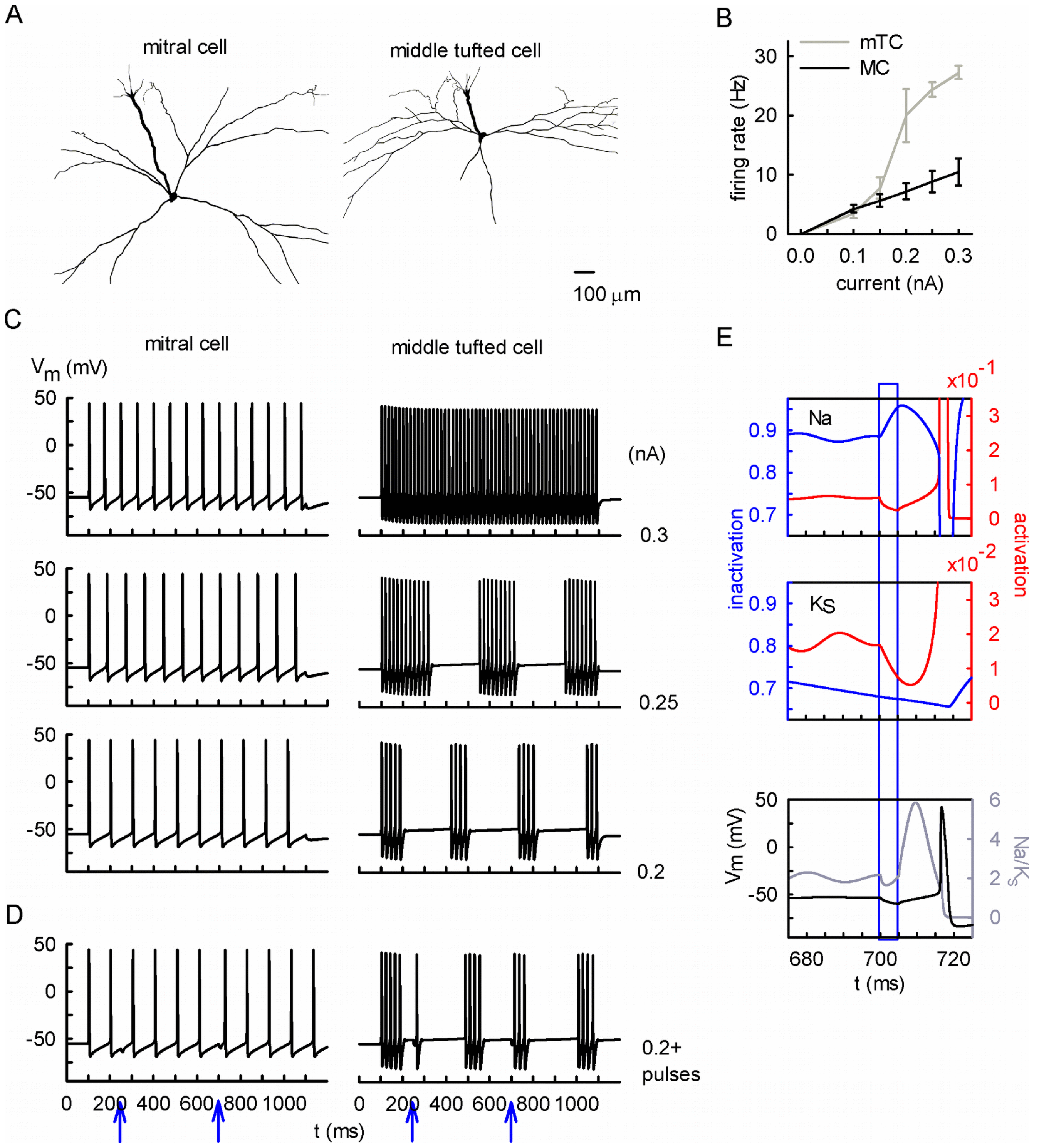

Figure S1. Different firing patterns in mitral and middle tufted cells to injected current.

(A) Typical synthetic MCs (left) and mTCs (right).

(B) F-I curves for synthetic MCs (n=10) and mTCs (n=10).

(C) Different firing patterns of MCs (left) and mTCs (right) evoked by depolarizing somatic current injection.

(D) MC (left) and mTC (right) responses to a brief hyperpolarizing current pulse (blue arrows); only the mTC responds with a rebound burst.

(E) The dynamics of the ionic channels underlying the rebound burst in mTCs. The opening of inactivation (blue curves) and activation of (red curves) sodium (Na) (top), and slow-potassium (K_S) (middle) ion channels are reset by a hyperpolarizing pulse (blue box). The slower recovery of K_S activation (bottom) yields a high ratio (gray curve) between Na and K_S opening fractions, triggering a rebound spike (black curve).

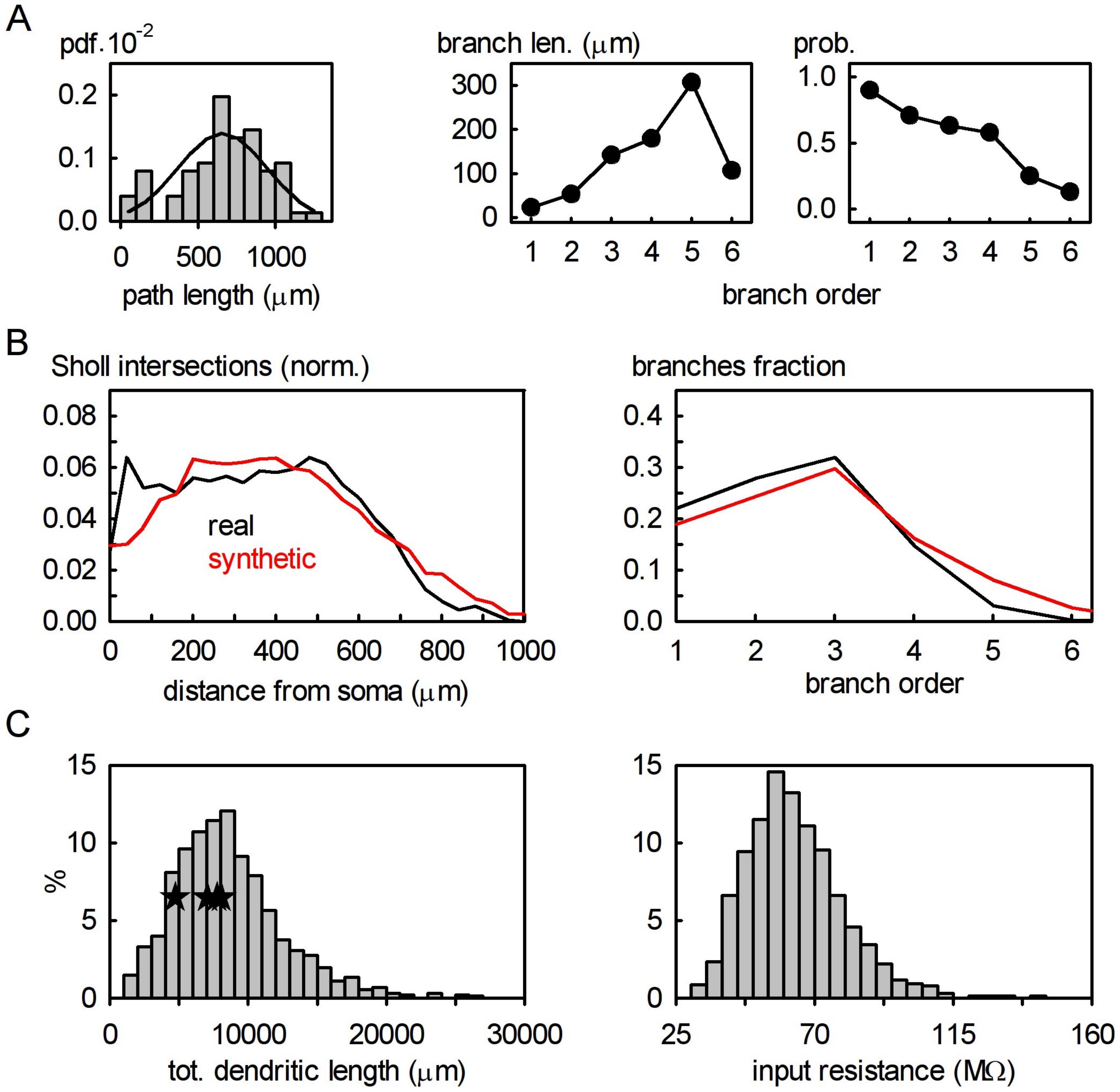

Figure S2. Generation of middle tufted cells for the circuit model.

(A) Statistical distribution of path length (left), branch length (middle), and branching probability (right).

(B) Sholl plot (left) and branch order (right) from experimental (black) and synthetic mTCs (red); they are statistically indistinguishable (Wilcoxon Signed Rank, $p = 0.949$ and $p = 1.0$, respectively).

(C) The distribution of the total dendritic length (left) in the synthetic cells resembles the total dendritic length observed in the experimental reconstructions (represented by the black stars, $n=6$); the distribution of mTCs input resistance (right; $n = 1{,}270$).

Table S1. Passive membrane properties in the model neurons.

	MC	mTC	sGC/dGC	dSAC
Specific membrane resistance (Ω /cm^2)	20,180	20,180	15,167	13,100
Specific membrane capacitance (μF·cm^2)	1	1	1.8	1
Axial resistance (Ω·cm)	150	150	70	70

Table S2. Active membrane properties in the model neurons.

	MC	mTC	sGC	dGC	dSAC
Na (S/cm^2)	0.04	0.04	0.04	0.027	0.04
K_A (S/cm^2)	0.004	0.004	0.004	0.004	0.004
K_{DR} (S/cm^2)	0.001	N/A	0.004	0.005	N/A
K_S (S/cm^2)	N/A	0.0055	N/A	N/A	N/A
